# Evaluation of lamina papyracea dehiscence with paranasal computed tomography

**DOI:** 10.1007/s00405-024-08538-8

**Published:** 2024-03-11

**Authors:** Muhammed Akif Deniz, Muhammed Tekinhatun

**Affiliations:** Department of Radiology, Dicle University Medical Faculty, Sur, Diyarbakır, Turkey

**Keywords:** Lamina papyracea, Dehiscence, Computed tomography, Paranasal sinus

## Abstract

**Introduction:**

The lamina papyracea is the thin line between the ethmoid sinus and the medial orbital wall. Knowledge of the presence of the lamina papyracea dehiscence (LPD) bears critical importance to prevent misdiagnosis of fractures at this level and to define the anatomy before sinonasal surgery, including Functional Endoscopic Sinus Surgery (FESS). The present study is therefore intended to determine the incidence of LPD in paranasal computed tomography, to identify its imaging characteristics in CT, and to compare with the literature.

**Materials and methods:**

The current study included patients who underwent paranasal CT scanning for any reason in our clinic between January 2018 and January 2022. Patients were evaluated in terms of age, gender, and presence of LPD. Patients with dehiscence were evaluated in terms of age, gender, dehiscence localization (right, left), tissue at the level of dehiscence, dehiscence size, and dehiscence grade.

**Results:**

1000 patients with a mean age of 32. ± 16.3 (min = 18-max = 79) were included in the study. 20 patients (2%) were found to have LPD. Of those with LPD, 14 (70%) were grade 1, 4 (20%) were grade 2 and 2 (10%) were grade 3. Again of those with LPD, 14 (70%) had LPD localized on the right and 6 (30%) had LPD on the left. In 12 (60%) of the patients with LPD, herniated tissue was detected. Among these patients with herniation, fatty tissue herniation was observed in 10 (83.3%) and medial rectus muscle herniation was observed in 2 (16.7%).

**Discussion and Conclusion:**

Comprehensive evaluation for and identification of LPD are very important before possible sinus surgery.

## Introductıon

The nasal cavities and paranasal sinuses are probably one of the most frequently varied areas of the human body. Due to their complex three-dimensional structure and the presence of many morphologic variations they possess, understanding these anatomical features is of great importance for sinus surgeons [1, 2].

The lamina papyracea is the thin line between the ethmoid sinus and the medial orbital wall. It usually has a very fine structure, hence the name papyracea. This structure prevents the transmission of infection and tumor at the level of the ethmoid sinus to the orbital region. Thinning or interruption in this area can lead to extension of ethmoid sinusitis and tumor into the orbital region. In these cases, orbital cellulitis, orbital edema-infection may occur, as well as blindness in advanced cases [3].

Dehiscence is defined as a focal or diffuse thinning and indistinguishability of the bone structure surrounding a tissue. Especially during surgery, dehiscence in vascular structures may cause injuries and mortality [3]. Knowledge of the presence of the lamina papyracea dehiscence (LPD) bears critical importance to prevent misdiagnosis of fractures at this level and to define the anatomy before sinonasal surgery, including Functional Endoscopic Sinus Surgery (FESS), as well as to enhance the accuracy rate in interpreting when evaluating sinus pathologies such as infection and tumor infiltration. It is therefore important for radiologists to be aware of this anatomical variant and to mention its presence in their reports. Since Computed Tomography (CT) helps obtain an elaborative image of the anatomy and bony structures of the paranasal region, it is frequently used for pathologies in this region.

The present study is therefore intended to determine the incidence of LPD in paranasal computed tomography, to identify its imaging characteristics in CT, and to compare with the literature.

## Materials and methods

Our study was designed as a retrospective study and approved by the local ethics committee (Number: 243, Date: 09.06.2022). It included 1000 patients (over 18 years old) who underwent paranasal CT scanning for any reason in our clinic between January 2018 and January 2022 and in whom the lamina papyracea could be visualized completely. Patients were evaluated in terms of age, gender, and presence of LPD. Patients with dehiscence were evaluated in terms of age, gender, dehiscence localization (right, left), tissue at the level of dehiscence, dehiscence size, and dehiscence grade.

Wall discontinuity at the level of Lamina Papyracea is considered as LPD. Grade 1 dehiscence is defined as less than 1/3 of the wall affected, grade 2 dehiscence is defined as 1/3 to 2/3 of the wall affected, and grade 3 dehiscence is defined as more than 2/3 of the wall affected by the discontinuity [4–8]].

### CT and examination protocol

All examinations were performed with a 64-detector CT (Philips Brilliance 64 Channel, Philips Healthcare, Eindhoven, The Netherlands). (The parameters used in the imaging protocol are: 120 kVp, 300mAs, 1 mm section thickness, 0.5 pitch and 220 mm field of view.) All images were sent to the Radiologic Imaging and Archiving System (PACS) and multiplanar images were created and evaluated. All images were evaluated by two radiologists with at least 5 years of experience in interpreting the images of head and neck regions. In cases of images where the two radiologists provided different opinions, those images were evaluated by them again together to reach a consensus. In patients with multiple images, only one image without artifacts and with clearly distinguishable borders of the lamina papyracea was included in the study.

In our study, while paranasal CT images were evaluated from the PACS system, the patients' admission to the outpatient clinic, epicrisis, and surgery notes were also evaluated from the hospital information management system. Patients with a history of trauma, fracture, or surgical operation in their epicrisis or outpatient clinic records, and patients for whom clear information could not be obtained on this subject, images with artifacts, images where the structures of the lamina papyracea cannot be clearly evaluated, and images of patients with disruption of the normal anatomical structure due to trauma, fracture, mass, or surgical operation were not included in the study.

### Statistical analysis

The analyses were assessed in SPSS (Statistical Package for Social Sciences; SPSS Inc., Chicago, IL) 22 software package. Descriptive data were presented as n (%) for the categorical data and mean ± standard deviation (Mean ± SD) for the continuous data. Chi-square analysis (Pearson Chi-square) was employed to perform an intergroup comparison of the categorical variables. Normality of the distribution of the continuous variables was tested using Kolmogorov–Smirnov test. Additionally, Mann–Whitney U test was used for the comparison of paired groups. In the analyses, p < 0.05 was considered statistically significant.

## Results

**Fig. 1:**
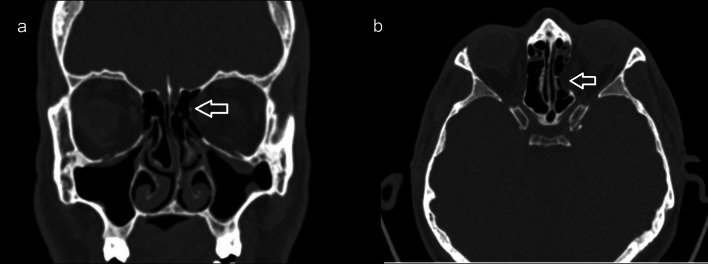
45-Year-old male patient with left (arrow) lamina papyracea dehiscence on coronal (**a**) and axial (**b**) paranasal CT image

**Fig. 2:**
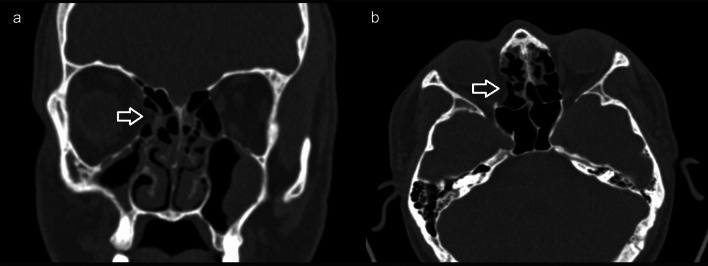
52-Year-old female patient with lamina papyracea dehiscence on the right (arrow) on coronal (**a**) and axial (**b**) paranasal CT image

Thousand patients with a mean age of 32. ± 16.3 (min = 18-max = 79) were included in the study. Forty-eight percent of the patients were female and 52% were male. 20 patients (2%) were found to have LPD. Of those with LPD, 14 (70%) were grade 1, 4 (20%) were grade 2 and 2 (10%) were grade 3. Again of those with LPD, 6 (30%) patients had LPD on the left (Fig. 1) and 14 (70%) patients had LPD localized on the right (Fig. 2). In 12 (60%) of the patients with LPD, herniated tissue was detected. Among these patients with herniation, fatty tissue herniation was observed in 10 (83.3%) and medial rectus muscle herniation was observed in 2 (16.7%). In addition, the location of dehiscence was posterior in 4 patients (20%), anterior in 8 patients (40%) and both anterior and posterior ethmoid sinus in the remaining 8 patients (40%). (Table 1).

**Table 1:** All characteristics of the patients included in the study

	Number (*n*)	%
Age, mean ± SD	31.6 ± 13.2	
Gender		
Female	480	48.0
Male	520	52.0
Dehiscence of lamina papyracea present		
Yes	20	2.0
No	980	98.0
Grade		
Grade 1	14	70.0
Grade 2	4	20.0
Grade 3	2	10.0
Dehiscence location		
Right	14	70.0
Left	6	30.0
Anterior ethmoid sinus	8	40.0
Posterior ethmoid sinus	4	20.0
Anterior and posterior ethmoid sinus	8	40.0
Presence of herniated tissue		
Yes	12	60.0
No	8	40.0
Type of herniated tissue		
Fatty tissue	10	83.3
Medial rectus muscle	2	16.7

The rate of LPD in men (3.1%) was significantly higher than that in women (0.8%) (*p* = 0.011). Furthermore, the mean age of patients with LPD was significantly higher than the mean age of patients without dehiscence (*p* = 0.004) (Table 2).

**Table 2:** Comparison of the demographic data of patients

	Female		Male		p*	Age	p**
	Number (*n*)	%	Number (*n*)	%		Mean ± SD	
Dehiscence							
Yes	4	0.8	16	3.1	**0.011**	40.7 ± 16.1	**0.004**
No	476	99.2	504	96.9		31.4 ± 13.0	
Localization							
Right	4	100.0	10	62.5	0.267	43.7 ± 18.0	0.444
Left	0	0	6	37.5		33.7 ± 8.1	
Herniated tissue							
Yes	2	50.0	10	62.5	0.648	45.3 ± 18.8	0.238
No	2	50.0	6	37.5		33.8 ± 7.6	

Bold indicates statistically significant

^*^Chi-square analysis

^**^Mann–Whitney *U* test was applied

Of those with right LPD, 71.4% were grade 1 and 28.6% were grade 2, while 66.7% of those with left LPD were grade 1 and 33.3% were grade 2. There was no significant difference between these statistical results (*p* = 0.063). Among those with herniated tissue, 66.7% were grade 1, 16.7% were grade 2, and 16.7% were grade 3, while 75% of those without left herniated tissue were grade 1 and 25% were grade 2. No significant difference was found in this term (*p* = 0.619) (Table 3).

**Table 3:** Comparison of the location of lamina papyracea dehiscence and the presence of herniated tissue by grade

	Grade 1		Grade 2		Grade 3		p*
	Number	%	Number	%	Number	%	
Localization							
Right	10	71.4	4	28.6	0	0	0.063
Left	4	66.7	0	0	2	33.3	
Presence of herniated tissue							
Yes	8	66.7	2	16.7	2	16.7	0.619
No	6	75.0	2	25.0	0	0	

^*^Chi-square analysis was performed

## Discussion

The lamina papyracea is the weakest spot of the medial orbital wall [3]. LPD is a variation of the paranasal sinuses defined as a defect of the medial orbital wall. It is considered a congenital variation that is usually asymptomatic. Dehiscence is characterized by protrusion of orbital fat, most often due to a defect in the wall of the anterior ethmoid sinus. The posterior border of the dehiscence usually tends to be the basal lamella, but the anterior border may vary [3]. Differential diagnosis with fracture-related dehiscence is important. Knowledge of this variation is highly important in planning the procedures to be performed on sinuses such as functional endoscopic sinus surgery (FESS). CT is important in the diagnosis and necessary in preoperative planning [4, 5].

The incidence of LPD has been reported between 0.76% and 10% in the literature [1, 2]. Jing Xu et al. [3] reported an incidence rate of 2.58% in a series of 893 CT scans, while another series of 1024 cases [6] reported an incidence of 6.5% and Kitaguchi et al. [7] reported an incidence of 1.9% in their study. In the series of 1000 paranasal CT scans included in our study, LPD was observed in 20 cases and the incidence was 2%.

LPD is divided into 3 grades according to the extent of herniation [6–8]. In the study by Xu et al. [3], grade 1 was the most common (69%) and grade 3 was the least common (8.70%). Han et al. [6] reported that grade 1 was the most common and grade 3 dehiscence was the least common in their study. Similar to the literature, grade 1 was the most common (70%) and grade 3 was the least common (10%) in our study.

Dehiscence of the lamina papyracea occurs usually unilaterally and in rare cases bilaterally. According to Li et al. [3], the incidence rates of dehiscence on right and left sides were similar and not statistically different. Similarly, Kaya et al. [9] found no statistically significant correlation between the dehiscence of right and left sides in their study. In our study, dehiscence was most commonly observed on the right side and grade 1 dehiscence was the most common among all the cases with dehiscence on right and left. In our study, no significant difference was observed between dehiscence localization and dehiscence grade.

Although orbital fatty tissue is most commonly observed at the level of dehiscence, orbital muscles can also be present in these areas. In many studies in the literature, orbital fatty tissue herniation was frequently encountered and it was rare to encounter orbital muscle herniation. Herniation tissue can involve intraocular muscles, especially the medial rectus [3, 7, 8]. In our study, herniated tissue was present in 60% of the patients with dehiscence, and orbital fatty tissue was the most common (83%) in patients with herniated tissue, consistent with the literature. In addition, no correlation was found between side and the presence of concomitant herniated tissue in our study.

Although not statistically significant, it has been reported that LPD increases with age in the literature [6, 8]. Han et al. [6] emphasize that the incidence of lamina papyracea dehiscence was quite low in the childhood age group and that it might tend to increase in later ages even if it was not statistically significant. In a study conducted by Açar et al. [8] using morphometric measurements, its incidence rate was reported to be statistically higher in men than in women. Kaya et al. found no statistically significant correlation between age and gender in their study [9]. In addition, although some studies in the literature emphasized that it was more common in men, no statistically significant difference was found between the sexes in these studies [1–3]. In our study, a significant correlation was found between increasing age and dehiscence incidence. We think that this is due to the changes that occur in bones with age. In addition, the frequency of LPD was found to be significantly higher in men than in women in our study.

Moulin et al. [1] found that dehiscence was limited to the anterior ethmoid sinus in a study of 783 patients. Kitaguchi et al. [7] also reported cases of dehiscence limited to the anterior ethmoid sinus. In a study by Xu et al. [3] with 893 patients, dehiscence was mostly observed at the posterior ethmoid sinus level. In our study, similar to the study of Xu et al., dehiscence was mostly observed at the posterior ethmoid sinus level.

The present study has certain limitations. The most important limitation of our study is its retrospective design. The relatively small number of patients and the inability to study the patients for any potential correlation they might have with the operative/surgical appearance are also among the limitations of our study.

## Conclusion

Prevention is the main way to best manage potential complications. Therefore, it is critical for the sinus surgeon to understand not only the "standard" anatomy but also the LPD evaluated in this study. Thanks to modern imaging techniques, anatomical differences can be detected and possible risks can be predicted in advance. Comprehensive evaluation and identification of LPD is very important in terms of possible complications before sinus surgery. In our study, a significant relationship was first found between age, male gender, and LPD. More work may be needed on this subject. For a comprehensive evaluation and appropriate treatment of LPD, it is important to recognize it by the radiologist, followed by treatment planning by an Otolaryngologist (ENT) or sinus surgeon.

## Data Availability

Not applicable.
